# Science deserves to be judged by its contents, not by its wrapping: Revisiting Seglen's work on journal impact and research evaluation

**DOI:** 10.1371/journal.pone.0174205

**Published:** 2017-03-28

**Authors:** Lin Zhang, Ronald Rousseau, Gunnar Sivertsen

**Affiliations:** 1 Dept. Management and Economics, North China University of Water Resources and Electric Power, Zhengzhou, China; 2 Centre for R&D Monitoring (ECOOM) and Dept. MSI, KU Leuven, Belgium; 3 Dept. Mathematics, KU Leuven & Fac. of Social Sciences, University of Antwerp, Belgium; 4 Nordic Institute for Studies in Innovation, Research and Education, Oslo, Norway; Max Planck Society, GERMANY

## Abstract

The scientific foundation for the criticism on the use of the Journal Impact Factor (JIF) in evaluations of individual researchers and their publications was laid between 1989 and 1997 in a series of articles by Per O. Seglen. His basic work has since influenced initiatives such as the San Francisco Declaration on Research Assessment (DORA), the Leiden Manifesto for research metrics, and The Metric Tide review on the role of metrics in research assessment and management. Seglen studied the publications of only 16 senior biomedical scientists. We investigate whether Seglen’s main findings still hold when using the same methods for a much larger group of Norwegian biomedical scientists with more than 18,000 publications. Our results support and add new insights to Seglen’s basic work.

## Introduction

The average citation impact of a journal is only a weak predictor of the citation impact of individual publications in that journal because, among other aspects, article citedness tends to be highly skewed among publications [1, 2]. Nevertheless, the Journal Impact Factor (JIF) is widely used for the evaluation of individual researchers and their articles. This practice has recently influenced in a series of well-organized reactions from scientific communities. First came the San Francisco Declaration on Research Assessment [3], which was initiated by the American Society for Cell Biology and now has more than 13,000 signees across the world. Then, published in *Nature* in April 2015 by experts in bibliometrics and research evaluation, came the Leiden Manifesto for research metrics, an annotated list of ten principles to guide research evaluation [4]. A few months later appeared *The Metric Tide* report [5], which provided the Higher Education Funding Council for England with an independent review on the role of metrics in research assessment and management.

All these documents agree with Eugene Garfield, who invented the JIF fifty years ago, in warning against the abuse of the indicator in research evaluation [6]:

*It would be more relevant to use the actual impact (citation frequency) of individual papers in evaluating the work of individual scientists rather than using the journal impact factor as a surrogate. The latter practice is fraught with difficulties, as Seglen and others have pointed out*.

Per O. Seglen is one of Norway’s most prolific biomedical scientists. In the late eighties, he was subjected to an evaluation in which the 16 research groups within his organization were ranked according to the impact of the journals in their publication lists. As the results seemed strange, Seglen engaged himself in studying the scientific basis for the evaluation method and soon became an influential bibliometric researcher himself. “From bad to worse: Evaluation by journal impact” was the title of the first international publication resulting from his reflections [1]. After more thorough investigations into “The skewness of science” [7] and the “Causal relationship between article citedness and journal impact” [8], his most influential article appeared in the *BMJ*: “Why the impact factor of journals should not be used for evaluating research” [9]. In this article he alludes at people who judge science by its wrapping (the journal) rather than by its contents. Cited more than two thousand times (Google Scholar), it has also influenced policy, including the three documents mentioned above.

Seglen’s pioneering work on the relation between article citedness and journal impact, as well as his conclusions concerning research evaluation, have since been supported by numerous studies [10–17]; see also Waltman [2] for the most recent review. Nevertheless, Seglen’s work has never been replicated. It was based on the publications originating from only 16 principal investigators. Thirty years ago, a sample of this size was not considered small in bibliometric research since the data had to be recorded by hand from the printed volumes of the Science Citation Index.

Our intention here is not to replicate, in the strict sense of the word, but to investigate whether Seglen’s main findings still hold when using the same methods for a much larger group of scientists. One of our three research questions is related to the general phenomenon that Seglen studied: *How skewed is article citedness*? Logically, the correlation between article citedness and journal impact should increase when studying a larger number of items. Hence, the second question is: *What is the correlation between article citedness and journal impact in a large population of authors and articles*?

**Fig 1:**
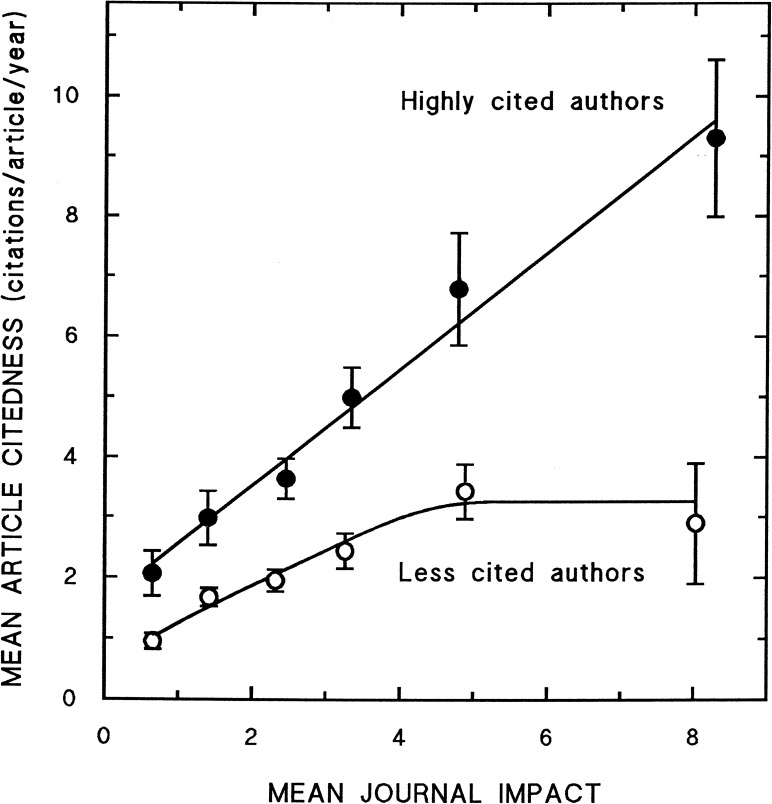
Correlation between journal impact and article citedness: a comparison between highly cited and less cited authors ([8], p.9, Fig 13). We thank Per Seglen for forwarding us his original figure.

Our third research question takes the perspective of the individual researcher: *Is there a benefit–in terms of received citations–of publishing in a journal with higher impact*? The obvious answer is: yes, on average, i.e. in the statistical sense, and in the short-term. The real question is: is it worth trying? Seglen [8] observed that when dividing the set of authors into two halves, the highly cited ones and the less cited ones, the highly cited authors are more cited than the journal’s impact factor would predict while the less cited authors are less cited than the JIF would predict. Most remarkably, while both groups’ mean article citedness increases with the mean journal impact, this increase stops for the less cited group when publishing in the journals with the highest impact. One could say that when this less cited group happens to have an article accepted in the highest impact journals, they do not come close to the journal impact–on average—as for other journals, but they reach a ceiling as illustrated in Seglen’s Fig 13 [8], reproduced here as Fig 1.

A recent study demonstrated that there is no certain benefit of publishing in a journal with a higher JIF instead of another with a lower value. The benefit grows only slowly as a function of the ratio of JIF values [18]. Normally, the question if publishing in a high-impact journal automatically leads to more citations can only be answered in the statistical sense, but Perneger [19] and Shanahan [20] found a case where the same text was published in different journals, namely the case of biomedical reporting guidelines that are published simultaneously in several journals to encourage wider adoption and dissemination. Both studies found that the number of citations received by the same guideline was moderately to strongly correlated to the JIF of the journal in which it was published.

Since Seglen’s time, several alternatives to the JIF have been proposed among which the SNIP index is the best known [21, 22]. These indicators of journal impact are also influenced by the skewness of citation distributions and hence have the same limitations with regard to the evaluation of individual articles.

To sum up, we investigate three questions concerning the skewness of article citedness (question 1), the correlation between article citedness and journal impact (question 2), and the possible benefit for citedness of publishing in a journal with higher impact (question 3), by using Seglen’s methods on a much larger set of data than he was able to establish thirty years ago.

## Data

Seglen studied 16 principal investigators in biomedical research at one Norwegian institution as authors of a total of 907 publications. In order to have a group of authors working in similar fields as Seglen’s we only include Norwegian scientists active in the biomedical sciences, resulting in 899 researchers working at–in principle all—Norwegian institutions. The total number of unique publications is 18,280. Their citations have been counted until the end of 2015.

The data source is Clarivate Analytics’ (formerly Thomson Reuters’) *National Citation Report for Norway* (NCR), a database with a selection of all Web of Science (WoS) records with a minimum of one institutional affiliation in Norway. Only records of the document type “Article” (original research articles) are included in our study. For the disambiguation of author names and affiliations, we selected only those records that could be matched to the CRISTIN database, Norway’s national current research information system, which has a complete representation of all published output at Norwegian universities, university colleges, hospitals and independent research institutions. The data are thereby linked to real persons and institutions. Biomedical researchers were defined as those active with at least ten publications within a large set of biomedical journals, using WoS journal categories. Among the 899 researchers in the database, 564 met these requirements. These are the scientists studied further on. Publications by the same researcher in other journals (not in the original set of biomedical journals) were included after the researchers had been identified. The full bibliography of each researcher within WoS and the period of study are thereby represented in our data.

Data for JIFs were retrieved from the Journal Citation Reports, another product from Clarivate Analytics that is based on the WoS. Since the latest volume year of 2-year JIFs we could retrieve was for 2015, and the earliest was 1994, we restricted our data collection from the NCR to publication years between 1992 and 2013. This data set also facilitates a three-year citation window (beginning in the publication year) for citedness analysis.

For each record in the NCR, Clarivate Analytics has added indicators based on the same methodology as in the online bibliometric toolbox *InCites*. Building on this methodology, we adopt the following indicators for this study:

*Number of Citations (NC)*: the number of received citations in a three-year citation window, starting from the publication year of an article. Concretely, if an article is published in the year Y, we consider the received citations in the year Y, Y+1, Y+2. This is the first measure of citedness in our study.

*Relative Citedness (RelCit)*: The second measure of citedness is a relative citation measure. According to the information provided by *InCites*- Clarivate Analytics, the *Relative Citedness* (*RelCit*) of a document is calculated by dividing the actual count of citing items by the expected number of received citations for documents of the same document type (article, in our case), year of publication and subject area. When a document is assigned to more than one subject area an average of the ratios of the actual to expected citations is used. Here the term ‘subject area’ refers to the WoS journal category of the article. For articles published in multidisciplinary journals, *InCites* uses a paper per paper assignment based on reference analysis. In this study, the subject field expected citation rate (baseline) is calculated over the period beginning in the year of publication and ending in 2015. This *Relative Citedness* is called Category Normalized Citation Impact (CNCI) in *InCites*.

Using the following notations: e = the expected citation rate or baseline, c = times cited, f = the field or subject area, t = year, d = document type, n = the number of areas a paper is assigned to, *RelCit* is defined as follows:

1. for a single article assigned to one subject area, *RelCit* is defined as:
  $$RelCit=\frac{c}{e_{ftd}}$$ (1)
2. for a single article assigned to n subject areas, *RelCit is*:
  $$RelCit=\frac{\sum_{j=1}^{n}\frac{c}{e_{f(j)td}}}{n}=\frac{\frac{c}{e_{f(1)td}}+\frac{c}{e_{f(2)td}}+\cdots +\frac{c}{e_{f(n)td}}}{n}$$ (2)

*Relative Citedness* can be considered an unbiased indicator of citation impact irrespective of age, subject focus and document type. A *Relative Citedness* value of 1 represents performance at par with world average, values above 1 are considered above average and values below 1 are considered below average.

Source:http://ipscience-help.thomsonreuters.com/inCites2Live/indicatorsGroup/aboutHandbook/usingCitationIndicatorsWisely/normalizedCitationImpact.html

*Journal Impact Factor (JIF)*: The Clarivate Analytics 2-year journal impact factor. Concretely, if an article is published in the year Y, we consider the JIF of its journal in the year Y+2. The *Number of Citations (NC)* of the target article will thus contribute to the *JIF* of its journal.

## Methods

As our database consists of authors, we have a complete data set (with respect to the WoS) for each author. When studying articles we de-duplicated records as articles can be co-authored by more than one Norwegian biomedical researcher.

As already mentioned in the data part, the notion of article citedness is operationalized in two ways:

1. *Number of Citations (NC)*: the received citations in three-year citation window, starting from the publication year of an article;
2. *Relative Citedness (RelCit)*: the relative citation impact normalized by subject field, publication year and publication type; citation windows always end in 2015.

When comparing citedness and the journal impact factor for the whole database, we calculated the Pearson and the Spearman correlation coefficients between Citedness values and the standard JIF as provided by Clarivate Analytics (two years after publication).

To answer our third research question (the benefit for citedness of publishing in high impact journals), we calculated correlations between JIF and Citedness for individual authors and tried to reproduce Seglen’s Fig 13 [8] by dividing the set of authors into highly cited and lower cited groups.

### A preliminary research question: What is the correlation between different measures of article citedness?

We stated that we operationalized the notion of article citedness in two ways, namely, the *Number of Citations* and *Relative Citedness*. The first measure is the actual citations in a three-year citation window, and the second one is the relative citation impact normalized by subject field, publication year and publication type. Note that citation windows for the latter measure always end in 2015, for all articles published in 1992–2013 in our data set. Before coming to the essential research questions we first answer the above mentioned preliminary question. We will show that these two different measures have a high correlation and hence expect that the answers to the main research questions will be independent from the specific article citedness measure. (We will nevertheless perform all investigations for the two measures of citedness.)

**Fig 2:**
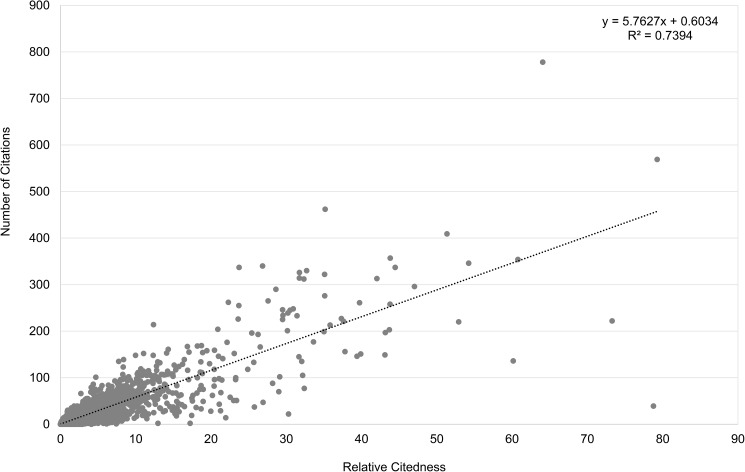
Relation between *Number of Citations* and *Relative Citedness* for all articles (1992–2013). Data from Clarivate Analytics’ National Citation Report for Norway.

Fig 2 displays the overall distributions of the two citation measures. The *Pearson correlation* between the two citedness measures over all articles is 0.90, indicating a high relationship between the actual received citations in a three-year window and the normalized citedness compared to the field standard. One outlier article which was published in 1994 is removed from the correlation calculation. This particular article had received 44 citations in a three-year citation window (1994–1996), while the total number of received citations amounted to 7,417 by the end of 2015. The *Relative Citedness* is thus as high as 201.82. Without removing the outlier, the Pearson correlation between the two citedness measures over all articles is 0.84. The Spearman correlation between the two citedness measures over all articles is 0.81.

**Fig 3:**
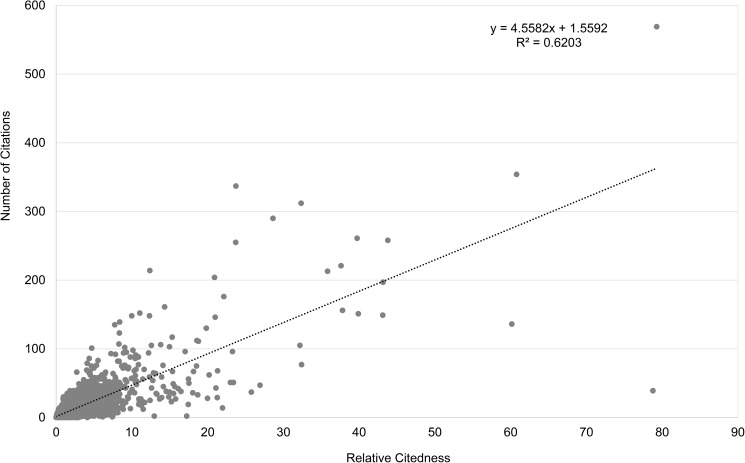
Relation between *Number of Citations* and *Relative Citedness* for articles published in 1992–2006. Data from Clarivate Analytics’ National Citation Report for Norway.

The question that arises here is whether the received number of citations in a three-year window can predict long-term citedness. For this investigation, we only include articles published in the period 1992–2006. For the calculation of *Relative Citedness* the citation windows always end in 2015. Hence we have a 10-year citation window for the youngest articles, and a 24-year window for the oldest ones. The *Pearson correlation* between the two citedness measures for articles published in 1992–2006 is 0.80, showing an obvious positive relation (Fig 3). The same outlier article with 7,417 citations by 2015 is removed. Without removing this article the Pearson correlation becomes 0.67. The Spearman correlation between the two citedness measures for all articles published in 1992–2006 is 0.73. It seems that the actual citations in 3 years can largely forecast future citation success; however, this correlation does not hold for each individual article. Some exceptional points can easily be observed in Figs 2 and 3: some articles received an extremely high immediate response (in 3 years), but had not achieved a high score for *Relative Citedness* in the long run. As a contrast, a few publications are only recognized over a long period of time. In order to have a better visualization, another two outlier articles with *Relative Citedness* higher than 100 are not shown in Figs 2 and 3. These results are in accordance with studies of the citation history of scientific articles [23,24,25,26,27].

**Fig 4:**
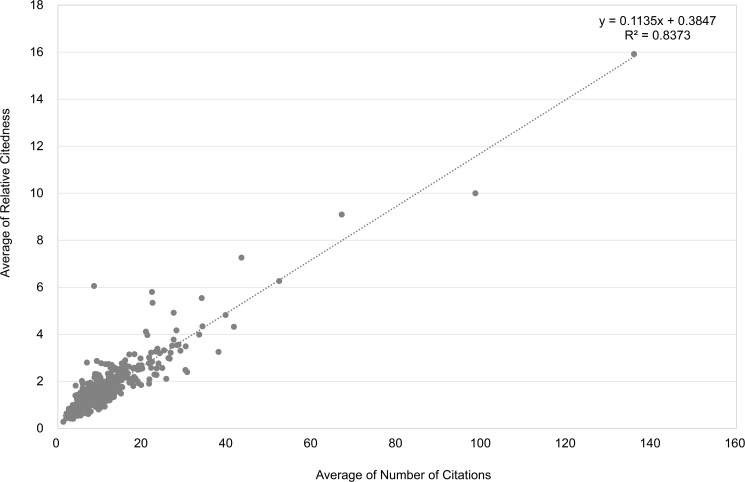
Relation between average values of *Number of Citations* and *Relative Citedness* for 564 authors with at least 10 publications. Data from Clarivate Analytics’ National Citation Report for Norway.

Next we consider citations on the author level. We recall that to obtain statistically reliable results on the author level, only authors having at least 10 articles in the underlying period are taken into consideration, which results in 564 most productive researchers. The *Pearson Correlation* between average values of *Number of Citations* and *Relative Citedness* for all 564 authors is 0.92. Fig 4 shows the corresponding distribution. One outlier author who published the above-mentioned outlier article (with 7,417 citations) is removed from the correlation calculation and from Fig 4. Without removing this author the Pearson correlation is 0.79. The corresponding Spearman correlation is 0.84. The two kinds of article citedness, although based on different methods (actual citation number vs. normalized citedness), and different citation windows (fixed three-year window vs. citation windows ending in 2015), have a high correlation on the author level. For this reason we do not expect large systematical differences when using the two different citedness measures. In general, active authors–remember that all these authors have at least ten publications in the biomedical journals- who receive more citations in a three-year window are also those having higher citedness in the long run, although this does not apply for each individual author.

### Main results 1: How skewed is article citedness?

**Fig 5:**
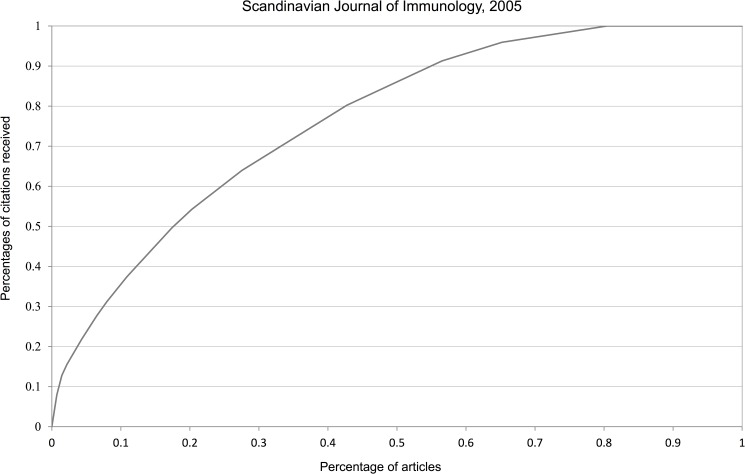
Lorenz curve of journal with the largest Gini coefficient (0.532).

**Fig 6:**
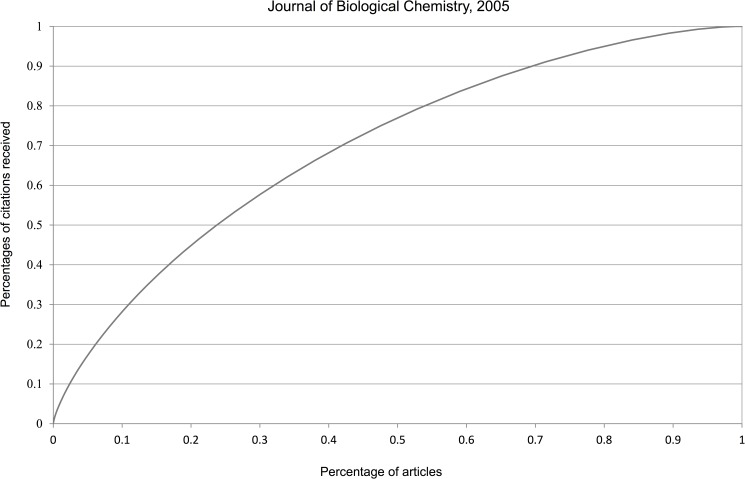
Lorenz curve of journal with the smallest Gini coefficient (0.395).

It is well-known that most informetric data are right-skewed. The many Lotka-type studies published in informetric articles bear testimony of this fact, see e.g. [28, 29]. Hence we just illustrate citation skewness by some journals which are among the most-used by the Norwegian scientists we study: the *Journal of Biological Chemistry* (JBC), the *International Journal of Cancer* (IJC) and the *Scandinavian Journal of Immunology* (SJI). We use two separate publications years: 2000 and 2005 and a citation window of three years, including the year of publication. Documents are restricted to those of ‘article’ type and collected from the Web of Science. Since the six resulting curves are very similar we only show those with the largest and the smallest Gini coefficient (Figs 5 and 6).

Table 1 illustrates the inequality of received citations (in a 3-year period) among these six cases. Although these percentages are less concentrated than in the proverbial 80/20 rule, they still illustrate the fact that it is unfair that the 50% of articles that received together (roughly) 20% of all citations are treated on an equal footing as the 20% of articles who received together 50% of all citations. Inequalities similar, and actually more outspoken, than those for journals can be seen on author level. In Table 1 we added the corresponding results for the five authors with most publications (each with more than 300 publications) in our dataset.

**Table 1:** Inequality among received citations in the same journal (and same publication year); inequality among received citations by the same author; both for a 3-year citation window

Journal / author	Percentage of articles receiving 50% of all citations	Percentages of citations received by 50% of all articles
JBC 2000	19	82
JBC 2005	18	86
IJC 2000	22	80
IJC 2005	21	79
SJI 2000	23	78
SJI 2005	24	77
Author ID (3223)	8	89
Author ID (18090)	17	83
Author ID (24290)	16	86
Author ID (52928)	8	90
Author ID (17700)	7	90

### Main results 2: What is the correlation between article citedness and journal impact?

#### Results related to all articles

**Fig 7:**
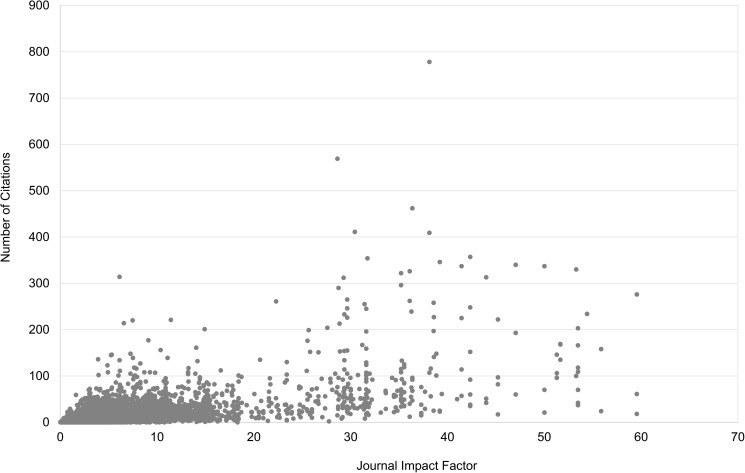
Relation between citedness (*Number of Citations in 3 years*) of individual articles and their corresponding journal impact. Data from Clarivate Analytics’ National Citation Report for Norway.

**Fig 8:**
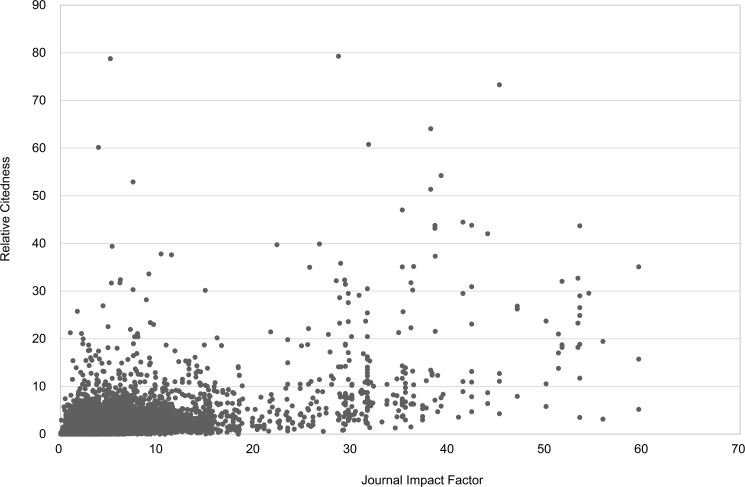
Relation between citedness (*Relative Citedness*) of individual articles and their corresponding journal impact. Data from Clarivate Analytics’ National Citation Report for Norway.

Table 2 presents Spearman correlations between JIF and the two different citedness measures. Seglen found a Pearson correlation of 0.41 for all articles under study. We have Spearman correlations equal to 0.52 and 0.40 (and Pearson correlations respectively equal to 0.51 and 0.43). These values are somewhat higher than Seglen’s, but still point to a moderate correlation. The obtained result is not surprising as each article can be considered as a sample from the journal distribution. Hence, values are by definition related, but because of the high skewness of citation distributions within journals one does not expect to find a high correlation. In Table 2 we further showed values for journals with a lower JIF (< 5) and for those with a higher one (> 10). Clearly correlations are significantly lower for the majority of articles published in journals with a lower JIF (73% of all articles under study). This confirms Seglen’s observation that, for the majority of articles, there is only a low correlation between JIF and article citedness. Figs 7 and 8 show that a moderately positive correlation can hide an uninformative set of data points. Three outlier articles (with Relative Citedness between 110 and 260) are removed from the figures in order to have a better visualization.

**Table 2:** Spearman correlations between JIF and article citedness. Data from Clarivate Analytics’ National Citation Report for Norway.

Article Citedness	All articles	JIF<5	JIF>10
*Number of Citations*	0.52	0.35	0.54
*Relative Citedness*	0.40	0.24	0.54

#### Results per author

We determined for each individual author (564 in total) the Spearman correlation between article citedness and the corresponding JIF. These correlations vary between -0.57 and 0.90 (for RelCit), and between -0.34 and 0.87 (for NR). Mean and median values are also presented in Table 3. Mean and median values show a moderate relation, but correlations can shift significantly from one author to another. We note that values for *Relative Citedness* have a larger range than those for *Number of Citations* and that mean and median values for *Relative Citedness* are smaller than those for *Number of Citations*. This remark corresponds with the observation made in [30] that normalized values lead to smaller correlations with the JIF than non-normalized ones.

**Table 3:** The Spearman correlation between article citedness and JIF for individual authors. Data from Clarivate Analytics’ National Citation Report for Norway.

Article Citedness	Lowest Value	Highest Value	Mean Value	Median Value
*Number of Citations*	-0.34	0.87	0.46	0.48
*Relative Citedness*	-0.57	0.90	0.40	0.41

**Fig 9:**
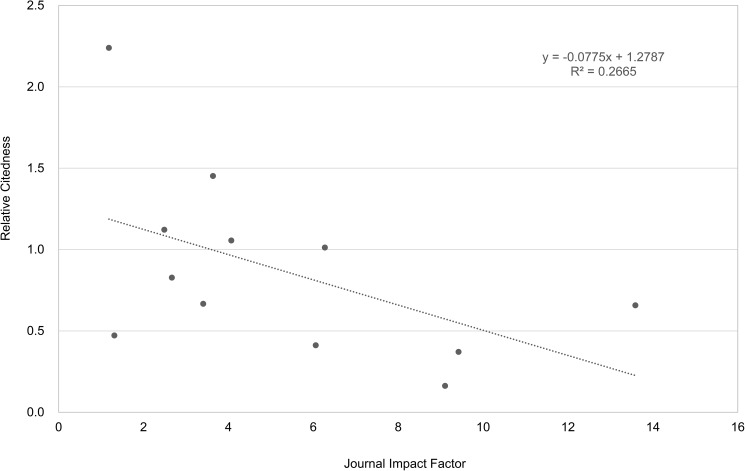
Relation between article citedness and corresponding journal impact for an author example (with the lowest correlation). Data from Clarivate Analytics’ National Citation Report for Norway.

**Fig 10:**
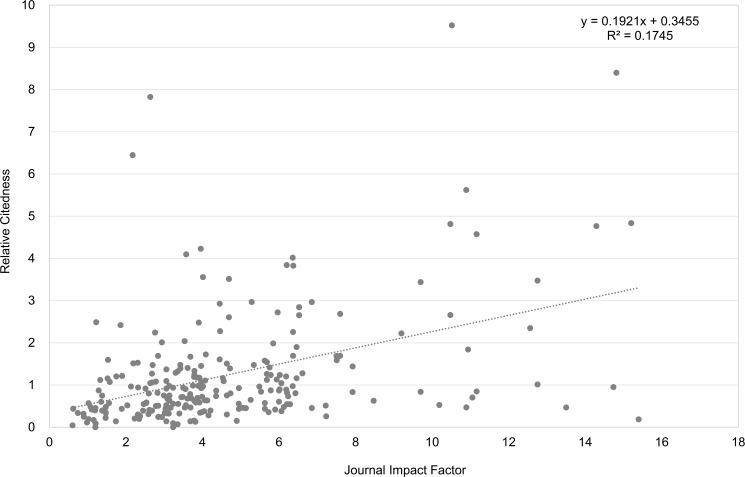
Relation between article citedness and corresponding journal impact for an author example (with a moderate correlation). Data from Clarivate Analytics’ National Citation Report for Norway.

Examples for authors with a negative moderate Spearman correlation (-0.57) and a positive moderate correlation (0.40) between their article *Relative Citedness* and corresponding JIF, are shown in Figs 9 and 10.

### Main results 3: What is the benefit for citedness of publishing in a journal with higher impact?

**Fig 11:**
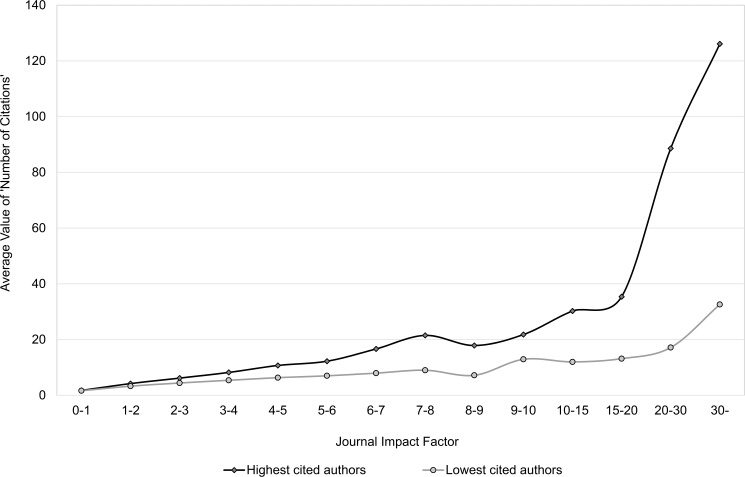
Relation between journal impact and article citedness (*Number of Citations*): a comparison between highly cited and less cited authors based on *Number of Citations*. Data from Clarivate Analytics’ National Citation Report for Norway.

**Fig 12:**
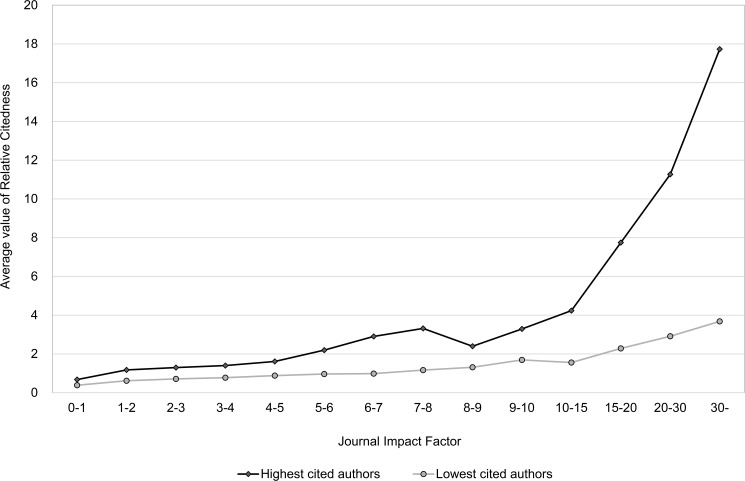
Relation between journal impact and article citedness (*Relative Citedness*): a comparison between highly cited and less cited authors based on *Relative Citedness*. Data from Clarivate Analytics’ National Citation Report for Norway.

Seglen divided the 16 authors in question into two groups: 8 highly cited authors and 8 lower cited authors. As we have much more authors we are able to make a better distinction between highly cited and lower cited authors. We ranked the 564 most productive authors according to their average values of citedness, respectively, *Number of Citations* and *Relative Citedness*, leading to four equally large groups each consisting of 141 authors. Group 1 stands for the highest cited authors, and Group 4 includes the authors with lowest citedness. The journal articles in each author group were pooled into fourteen journal impact cohorts, and the mean article citedness within each cohort was calculated. Figs 11 and 12 present the correlation between journal impact and mean article citedness, respectively for *Number of Citations* and *Relative Citedness*. Clearly, we could not exactly replicate Seglen’s findings. Yet, the difference between the groups is evident through all journal impact cohorts. Furthermore, despite the fact that both author groups’ average article citedness increases with the mean JIF, the citedness takes an eye-catching leap only for the highly cited group. When the less cited group happens to have publications appearing in the highest impact journals, the citedness of articles is only moderately influenced by the status of these journals.

The average value of *Spearman Correlation* between *JIF* and article citedness shows a clear decline from the more highly cited group of authors towards the less cited group (see Table 4). Consequently, the relation between article citedness and journal impact is even weaker for the less cited ‘normal’ majority of authors.

**Table 4:** The average value of ‘Spearman Correlation between *JIF* and article citedness’ for individual authors in different groups based on according article citedness [Data from Clarivate Analytics’ National Citation Report for Norway].

	Mean value of ‘Spearman Correlation between *JIF* and article citedness’			
Article Citedness	Group 1	Group 2	Group 3	Group 4
*Relative Citedness*	0.50	0.40	0.38	0.32
*Number of Citations*	0.56	0.48	0.42	0.37

## Conclusions

We confirm Seglen’s observation that there is no consistent positive relationship between individual article citedness and the impact factor of the journal in which the article is published. Our study of a much larger population of researchers than Seglen was able to study thirty years ago yielded only a marginally higher correlation.

We think, but admit we did not provide a proof, that a combination of two factors may explain the observed moderate correlation: 1) Citedness distributions are skewed in all journals. Less cited articles and highly cited articles may appear in any journal. While most articles are seldom cited, the few highly cited ones more often appear in 'highly cited' journals. 2) It is not the journals that are cited. Authors and their articles are cited. Less cited authors do not gain much from publishing in the journals where the more cited articles more often appear. It is the highly cited authors that provide these articles, perhaps by being conscious about where they publish their more significant results (a suggestion also made in [30]). The journal itself is not doing the job with regard to citations. Although some journals are certainly more prestigious, attractive and selective than others, one should not infer the quality of the individual article from the status of the journal. Moreover, even if citations are taken as an indication of quality, the citation impact of a journal remains a weak predictor of the citation impact of each of its articles. Consequently, individual contributions should not be evaluated by where they are published.

We can add to Seglen’s results that the lack of consistent positive relationship is more evident among the majority of researchers with average or lower citedness than among the minority of highly cited authors, and furthermore, correlations are significantly lower for the majority of articles published in journals with a lower JIF.

We used two methods to operationalize citedness: one uses the absolute number of citations during a three-year citation window, close to Seglen’s approach, while the other one is a relative method with a variable citation window. It turned out that the main results do not depend on the exact method, supporting the robustness of Seglen’s observations.

None of our findings are contrary to the understanding that JIFs should not be used as performance measures of individual researchers and their publications. To this we add a point not mentioned by Seglen, namely that using JIFs for research evaluation automatically excludes publications in journals not covered by WoS, in conference proceedings or in edited books, whatever their impact on their fields.
